# Pathogen Stimulation of Interleukin-8 from Human Vaginal Epithelial Cells through CD40

**DOI:** 10.1128/spectrum.00106-22

**Published:** 2022-03-17

**Authors:** Patrick M. Schlievert, Samuel H. Kilgore, Andrea Benavides, Aloysius J. Klingelhutz

**Affiliations:** a Department of Microbiology and Immunology, Carver College of Medicine, University of Iowa, Iowa City, Iowa, USA; Emory University School of Medicine

**Keywords:** CD40, *Candida*, *Escherichia coli*, *Neisseria gonorrhoeae*, *Staphylococcus*, *Streptococcus*, chemokines, epithelial cells, superantigens

## Abstract

Many bacterial and fungal pathogens cause disease across mucosal surfaces, and to a lesser extent through skin surfaces. Pathogens that potentially cause disease vaginally across epithelial cells include Staphylococcus aureus, group A and B streptococci, Escherichia coli, Neisseria gonorrhoeae, and Candida albicans. We have previously shown that staphylococcal and streptococcal superantigens induce inflammatory chemokines from vaginal epithelial cells through the immune costimulatory molecule CD40 through use of a CRISPR cas9 knockout mutant and complemented epithelial cell line. In this study, we show that the potential vaginal pathogens S. aureus, group A and B streptococci, E. coli, an Enterococcus faecalis strain, and C. albicans in part use CD40 to stimulate interleukin-8 (IL-8) production from human vaginal epithelial cells. In contrast, N. gonorrhoeae does not appear to use CD40 to signal IL-8 production. Normal flora Lactobacillus crispatus and an Enterococcus faecalis strain that produces reutericyclin do not induce IL-8. These data indicate that many potential pathogens, but no normal commensals, induce IL-8 to help disrupt the human vaginal epithelial barrier through CD40, thus providing a potential therapeutic target for drug development.

**IMPORTANCE** Most bacterial and fungal pathogens cause disease across mucosal, and to a lesser extent, skin barriers with the help of induced chemokines from epithelial cells. In this study, we showed that potential vaginal pathogens Staphylococcus aureus, group A and B streptococci, some Enterococcus faecalis strains, Escherichia coli, and Candida albicans use the immune costimulatory molecule CD40 to induce the chemokine interleukin-8 production. In contrast, Neisseria gonorrhoeae does not use CD40 to stimulate interleukin-8. Normal flora lactobacilli and at least one E. faecalis strain do not induce interleukin-8.

## INTRODUCTION

Staphylococcus aureus, Streptococcus pyogenes (group A), Streptococcus agalactiae (group B), Escherichia coli, and Neisseria gonorrhoeae are all potential vaginal bacterial pathogens. Additionally, Candida albicans is a common vaginal fungal pathogen. In contrast, bacteria such as various lactobacilli are normal flora (1, 2). In one instance at least, a woman was identified with Enterococcus faecalis as the only vaginal microbe (3). This suggests this organism, in some individuals, is part of the normal vaginal microbiome.

Many of these pathogenic organisms often colonize mucous membranes and may colonize skin to initiate infections through these same surfaces (4, 5). The organisms are important vaginal mucosal pathogens but also cause infections across other mucosal surfaces. The Gram-positive pathogens (S. aureus, group A and B streptococci, and some strains of Enterococcus faecalis) also are causes of toxic shock syndrome (TSS) and many other kinds of infections through production of many virulence factors, including known or suspected superantigens (SAgs) (5–10). E. coli is the cause of 80% of urinary tract infections and a major cause of sepsis (11). Neisseria gonorrhoeae is the cause of gonorrhea, due to inflammation on mucosal surfaces including the vagina (12). C. albicans is the major cause of mucocutaneous candidiasis and is becoming a more common cause of sepsis (13).

All of these potential vaginal pathogens have been shown previously to be associated with elevated epithelial cell interleukin-8 (IL-8), a proinflammatory chemokine that attracts neutrophils to sites of infection (14–19). However, some of these studies evaluated epithelial cells not from the vagina but, instead, from the urinary tract (17), tonsils (16), or lungs (14). Other than through Toll-like receptor 2 (TLR2), other receptors for production of IL-8 were not determined. In one study, it was shown that some Streptococcus pyogenes strains (M1, for example) produce an IL-8 protease, leading to dampened IL-8 (16). One goal of the current studies was to assess possible additional receptors other than TLR2 used by potential vaginal pathogens to stimulate IL-8 production by human vaginal epithelial cells (HVECs). In prior studies, we evaluated the effect of S. aureus and its SAgs on both IL-8 and MIP-3α production (20). However, we later showed that MIP-3α is cleaved by S. aureus proteases (21). Thus, despite the 80-fold upregulation of IL-8 mRNA versus 400-fold upregulation of MIP-3α mRNA by S. aureus (20), the amount of MIP-3α protein was much reduced compared to IL-8; IL-8 was stable to S. aureus proteases (21). For this reason, the current studies focused on the effect of vaginal pathogens on IL-8 production.

We have shown that S. aureus SAgs dysregulate HVECs, causing thousands of genes to be up- or downregulated (20). The SAg TSS toxin-1 (TSST-1) altered by >2-fold nearly 2,400 genes in HVECs after 6 h of treatment (20). Many of the most highly upregulated genes were those encoding chemokines, such as IL-8 and MIP-3α, with MIP-3α expression, for example, being upregulated over 400-fold (20). The effects of TSST-1 and another SAg, staphylococcal enterotoxin B (SEB), on HVECs were mediated through SAg interaction with CD40, as demonstrated by CRISPR cas9 knockout studies of CD40 on these cells (22). There were estimated to be 5 × 10^4^ TSST-1 receptors per HVEC (20).

We have also examined the effect of TSST-1, SEB, and two streptococcal pyrogenic exotoxins (SPEs A and C) on primary human keratinocytes (23). Both TSST-1 and SEB caused significant up- and downregulation of nearly 5,800 genes for TSST-1 and 4,300 genes for SEB when tested against human keratinocytes (23). As expected, there was considerable overlap in genes affected by the two SAgs, including the upregulation of expression of many chemokine genes (23). With use of immortalized human keratinocytes, we have shown multiple chemokine genes were upregulated by these same two SAgs (23); among the upregulated genes were those that encoded chemokines, such as IL-8 and MIP-3α. For SPEs A and C, we examined only IL-8 production from human keratinocytes and showed that both SPEs stimulate production of the chemokine. Because of the high relatedness of SPE A to SEB, we have not examined the effect of this SAg on HVECs.

The purpose of the present study was to assess the potential use of CD40 as a receptor for vaginal mucosal pathogens. Our studies show that the majority of these organisms in part stimulate HVECs to produce proinflammatory IL-8 through CD40.

## RESULTS

All experiments in this study used replicates of three wells per treatment, and all experiments were performed a minimum of two times.

### Stimulation of IL-8 production by Gram-positive bacterial strains.

We utilized the following Staphylococcus aureus strains in the current studies: S. aureus MN8, a TSST-1 gene (*tst*) knockout mutant of MN8, and MNPE (24). S. aureus MN8 is a typical USA200 (CC30) menstrual TSS isolate with a mutation in the alpha-toxin structural gene, significantly downregulating production of alpha-toxin (25). S. aureus MNPE is also a USA200 (CC30) strain from a patient with postinfluenza TSS (24), but like other skin isolates, this organism has a wild-type alpha-toxin gene and produces typical amounts of alpha-toxin (approximately 50 μg/mL) in standard growth medium (Todd-Hewitt broth [THB]). Both S. aureus strains produce TSST-1 at approximately 10 to 20 μg/mL in THB under high aeration. The strains also produce β-toxin (approximately 100 μg/mL), which is characteristic of USA200 (CC30) strains, and the strains contain the enterotoxin gene cluster (EGC) of 6 SAgs (SEG, SE-like I, M, N, O, and U) that are usually produced in small amounts (26, 27). The EGC SAgs are encoded on an operon, and these SAgs are thought to be more important for colonization than production of TSS, based on their low level of production (26). The MN8Δ*tst* mutant is a clean mutant of MN8 in which the gene for TSST-1 production only was deleted. All three strains had stationary phases of approximately 7.0 × 10^9^/mL in Todd-Hewitt medium after growth for 24 h, at 37°C, with shaking (200 rpm).

**FIG 1:**
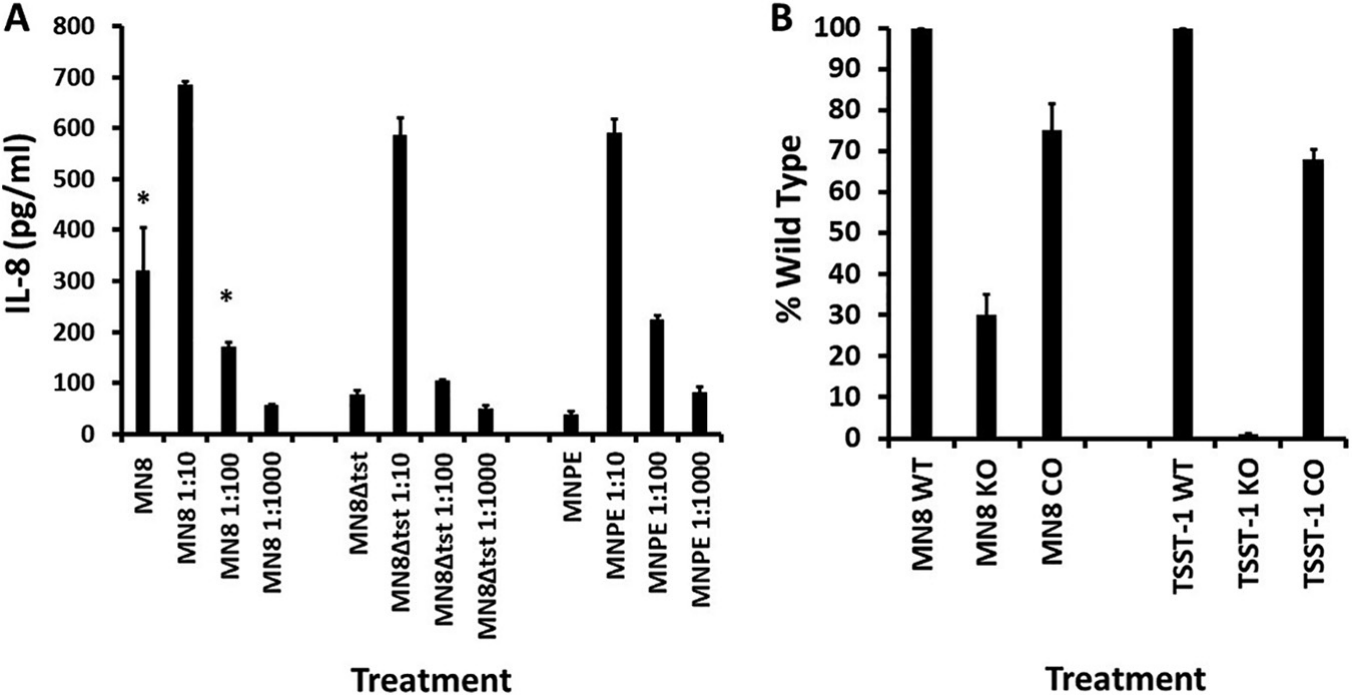
Wild-type (WT) human vaginal epithelial cells (HVECs), CD40 knockout (KO) HVECs, and CD40-complemented (CO) HVECs treated for 6 h in triplicate with various dilutions of S. aureus MN8, MN8Δ*tst*, and MNPE. (A) IL-8 ± standard deviations. *, significant mean differences between MN8 and MN8Δ*tst* with *P* < 0.0001. (B) Percent wild type of HVEC CD40 knockout cells (KO) and CD40-complemented HVEC knockout cells (CO) treated with the dose of MN8 (MN8 1:10) that gave the peak IL-8 response by wild-type cells. TSST-1 was used at 50 μg/mL as a control.

We performed dose-response experiments to assess the ability of dilutions of all three S. aureus strains (MN8, MN8Δ*tst*, and MNPE) to cause interleukin-8 (IL-8) production from immortalized HVECs (Fig. 1). It should be noted that even though the HVECs are immortalized, the cell phenotype is that of nonimmortalized HVECs (20, 22). All organisms induced the production of IL-8 (chemoattractant of polymorphonuclear leukocytes [PMNs]) as a function of CFU per milliliter of bacteria. Only data for IL-8 production, as representative of chemokine production by HVECs, are shown in this and all subsequent figures. We did key experiments previously to show that pathogens stimulated production of MIP-3α (20, 23), a chemokine that attracts both innate and adaptive immune cells to areas of damage.

All three S. aureus strains inhibited IL-8 production (Fig. 1A) at the highest bacterial concentration (7 × 10^9^ CFU/mL), likely due to killing of HVECs though production of cytotoxins (28). However, at 10-fold-lower doses of all three S. aureus strains, IL-8 was maximally produced. The amount of IL-8 was reduced by treatment with lower doses of all strains. At the three higher doses of MN8, more IL-8 production was observed compared to MN8Δ*tst.* This is consistent with TSST-1 contributing to IL-8 induction in HVECs as reported previously (20, 22).

We next evaluated the role of the immune costimulatory molecule CD40 in IL-8 production by S. aureus MN8, with TSST-1 as a positive control. We tested IL-8 production from wild-type HVECs, CRISPR cas9 CD40 knockout HVECs, and CD40-complemented cells in the CD40 knockout background. For purposes of ease of comparison, we show data as the percent stimulation compared to wild-type HVECs treated with 7 × 10^8^ CFU/mL, the maximum CFU for stimulation, and set at 100%. We also set the response of wild-type HVECs to 50 μg/mL of TSST-1 at 100%. All data are reported after 6 h of exposure to S. aureus or TSST-1 in keratinocyte serum-free medium (KSFM) at 37°C in the presence of 5% CO_2_. These HVEC incubation conditions were also used for all subsequent experiments.

For S. aureus MN8, 70% of the IL-8 production depended on the presence of CD40 (100% for wild-type HVECs versus 30% in CD40 knockout cells) as shown in Fig. 1B. In contrast, as expected and previously published (22), TSST-1 induction of IL-8 production depended 100% on CD40. For both S. aureus MN8 and TSST-1, there was significant restoration of IL-8 production in CD40-complemented cells, suggesting a lack of off-target CRISPR cas9 effects.

**FIG 2:**
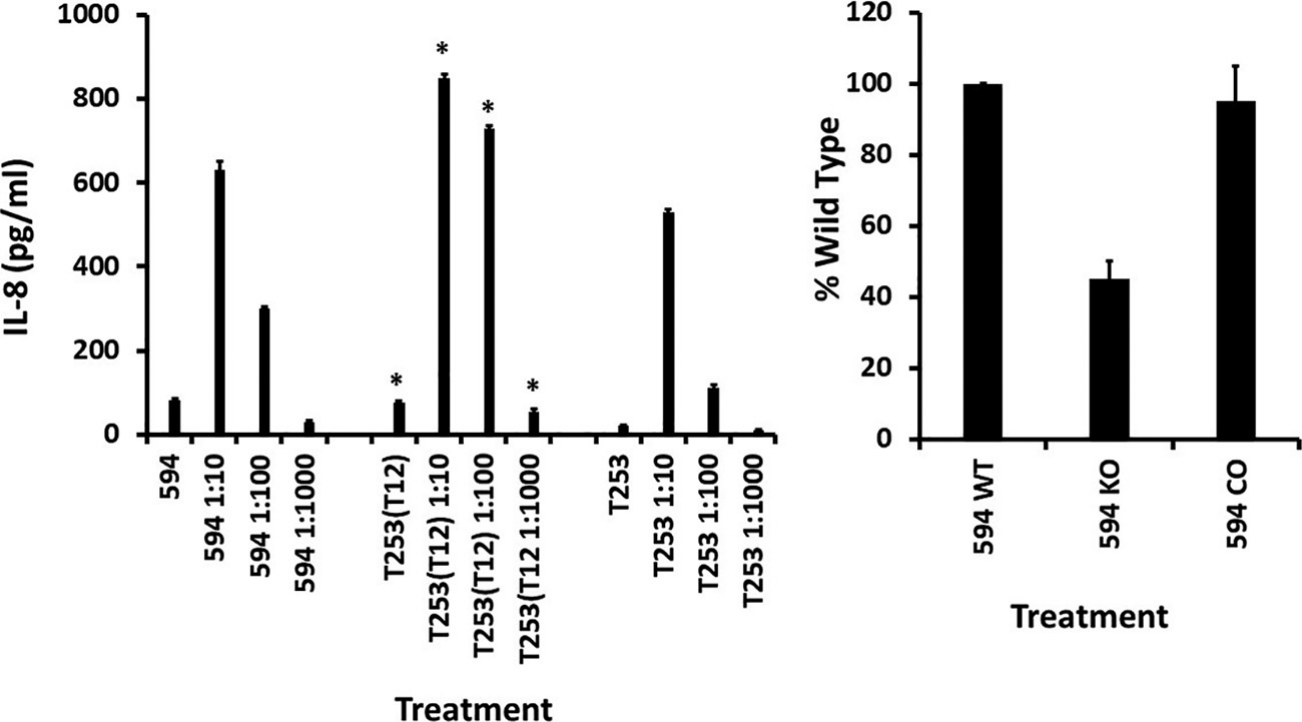
Wild-type (WT) human vaginal epithelial cells (HVECs), CD40 knockout (KO) HVECs, and CD40-complemented (CO) HVECs treated for 6 h in triplicate with various dilutions of group A streptococci 594, T25_3_(cured)T12, and T25_3_(cured). Left panel shows IL-8 ± standard deviations. *, mean differences between T25_3_(cured)T12 and T25_3_(cured) with *P* < 0.0001. Right panel shows percent wild type of HVEC CD40 knockout cells (KO) and CD40-complemented HVEC knockout cells (CO) treated with the dose of strain 594 (1:10) that gave the peak IL-8 response by wild-type cells.

Group A streptococcal strain 594 (29), T25_3_(cured)T12, and its phage-free counterpart T25_3_cured (30, 31) were evaluated in the same way as S. aureus, both in determination of dose response with HVECs for 6 h in production of IL-8 and in determination of percentage of the response due to interaction with CD40 (Fig. 2). Group A streptococcal strains 594 (29) and T25_3_(cured)T12 are typical beta-hemolytic group A strains that encode the superantigen streptococcal pyrogenic exotoxin A (SPE A), a SAg related to SEB, encoded by a gene on the bacteriophage T12 (30, 31). The T25_3_(cured)T12 strain, as opposed to the bacteriophage-free and thus SPE A-free strain T25_3_, causes TSS in a rabbit model (32). Both of the SPE A-positive strains produce approximately 5 μg/mL of the SAg in THB.

All three of the group A streptococcal strains grew to stationary phases of approximately 3 × 10^8^ to 4 × 10^8^ CFU/mL after overnight growth when incubated stationarily in a 5% CO_2_ incubator, at 37°C with a starting inoculum of approximately 10^7^ CFU/mL. The three strains, when added to HVECs in KSFM, inhibited IL-8 production at the 3 × 10^8^- to 4 × 10^8^-CFU/mL dose (Fig. 2, left panel), likely due to killing through production of cytotoxins (28, 33). When strains were diluted 10-fold, maximum IL-8 production was observed. Incubation with lower doses of all three organisms resulted in less IL-8 production by HVECs. Strain T25_3_(cured)T12 at all doses induced production of more IL-8 than was induced by incubation with T25_3_(cured). This may have been the result of production of SPE A, a SAg with known ability to induce IL-8 from human keratinocytes (23), by T25_3_(cured)T12 versus T25_3_(cured). However, both strains induced significant IL-8 production. When HVECs were treated with 100 μg/mL of SPE A alone, the SAg led to an average ± standard deviation of production of 255 ± 7.3 pg/mL of IL-8. This amount is similar to the response of wild-type HVECs to the SPE A-related SAg SEB (22). The IL-8 response to SPE A was eliminated in CD40 knockout cells (1.7 ± 1.7 pg/mL) and restored to 184 ± 4.8 pg/mL in CD40-complemented cells.

Also shown in Fig. 2 are the results of incubation of strain 594 with wild-type HVECs, CD40 knockout HVECs, and CD40 complemented in the knockout strain. The data were reported as percentage of IL-8 produced by wild-type HVECs in response to 3.0 × 10^7^ CFU/mL after 6 h of incubation, noting this is the dose of strain 594 that induced the greatest production of IL-8. The data showed that 60% of the IL-8 response of HVECs was due to direct or indirect interaction with CD40, as seen by a 60% reduction by the CD40 knockout strain. The CD40-complemented strains responded with nearly wild-type IL-8 production, indicating the reduced IL-8 response in the CD40 knockout strain was not the result of an off-target effect in the CRISPR cas9 knockout.

**FIG 3:**
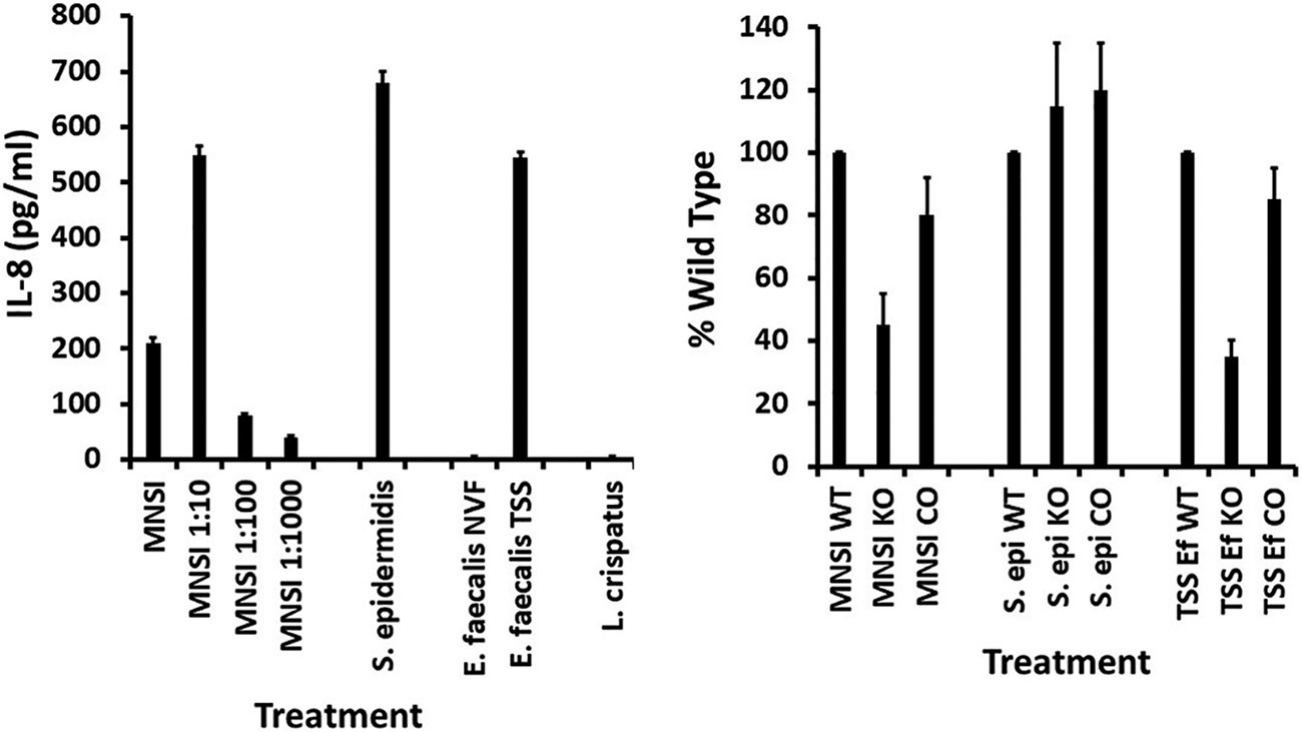
Wild-type (WT) human vaginal epithelial cells (HVECs), CD40 knockout (KO) HVECs, and CD40-complemented (CO) HVECs treated for 6 h in triplicate with various dilutions of group B streptococci (MNSI) and a single dose of S. epidermidis, two Enterococcus faecalis strains (NVF, normal vaginal flora; TSS, toxic shock syndrome), and a strain of L. crispatus. Left panel shows IL-8 ± standard deviations. Right panel shows percent wild type of HVEC CD40 knockout cells (KO) and CD40-complemented HVEC knockout cells (CO) treated with the dose of optimal strain that gave the peak IL-8 response by wild-type cells.

The same type of dose-response assay to a group B streptococcal strain associated with a neonatal TSS-like syndrome (9) (Fig. 3) was also performed. Also tested (Fig. 3) were a normal skin flora organism, Staphylococcus epidermidis (8 × 10^9^ CFU/mL) (a single dose since no cytolysin was produced by the strain), a human vaginal Enterococcus faecalis strain from a woman colonized with a pure culture of the organism (normal vaginal flora [NVF]; single dose of 5 × 10^8^ CFU/mL since no cytolysin was produced) (3), an E. faecalis strain from a patient with nonmenstrual TSS (single dose of 6 × 10^8^/mL since no cytolysin was produced), and a Lactobacillus crispatus strain isolated from the vagina of a healthy woman (single dose of 2 × 10^9^ CFU/mL since no cytolysin was produced).

The stationary phase of the group B streptococcal strain tested (MNSI) was 3.0 × 10^8^ CFU/mL after 24 h of growth at 37°C, without shaking, in a 5% CO_2_ incubator; the starting inoculum was approximately 10^7^ CFU/mL. Like both S. aureus and group A streptococci, the MNSI group B streptococcal strain caused high-dose inhibition of IL-8 production (Fig. 3, left panel), likely due to killing effects from cytotoxin production (28, 33). The peak for IL-8 production in HVECs was 3.0 × 10^7^ CFU/mL. Just over 50% of the response of HVECs to MNSI depended on CD40, as shown by the 50% reduction in IL-8 in the CD40 knockout strain (Fig. 3, right panel). The CD40-complemented strain showed significant restoration of the CD40 response.

The normal flora microbe S. epidermidis also caused significant induction of IL-8 production by wild-type HVECs (Fig. 3). There was no reason to evaluate the complemented HVECs for this organism since the CD40 knockout HVECs showed similar IL-8 induction as wild-type HVECs.

The normal vaginal flora strains of E. faecalis (3) and L. crispatus (2) did not induce IL-8 production by wild-type HVECs (Fig. 3, left panel). At least for the E. faecalis strain, we have provided evidence that this lack of IL-8 production likely resulted from reutericyclin production by the organism (3).

We also tested an E. faecalis strain from a male patient with nonmenstrual TSS for IL-8 production by HVECs. This male had TSS associated with a break in the skin. SAgs are the known causes of TSS. This E. faecalis strain has not thus far been tested for SAgs, but we know E. faecalis has the ability to produce SAg-like molecules (34). Additionally, we have shown that SAgs induce IL-8 from HVECs and human keratinocytes with induction of the CD40 pathway (20, 22, 23). The TSS E. faecalis strain induced IL-8 production by wild-type HVECs (Fig. 3, left panel). This induction depended approximately 70% on the presence of CD40, as the CD40 HVECs showed a 70% reduction in IL-8 compared to wild type (Fig. 3, right panel). The response was restored in the CD40-complemented HVECs. The NVF Enterococcus faecalis and L. crispatus were not tested in either the CD40 knockout strain or the CD40-complemented strain.

### Stimulation of IL-8 production by Gram-negative bacterial strains and Candida albicans.

**FIG 4:**
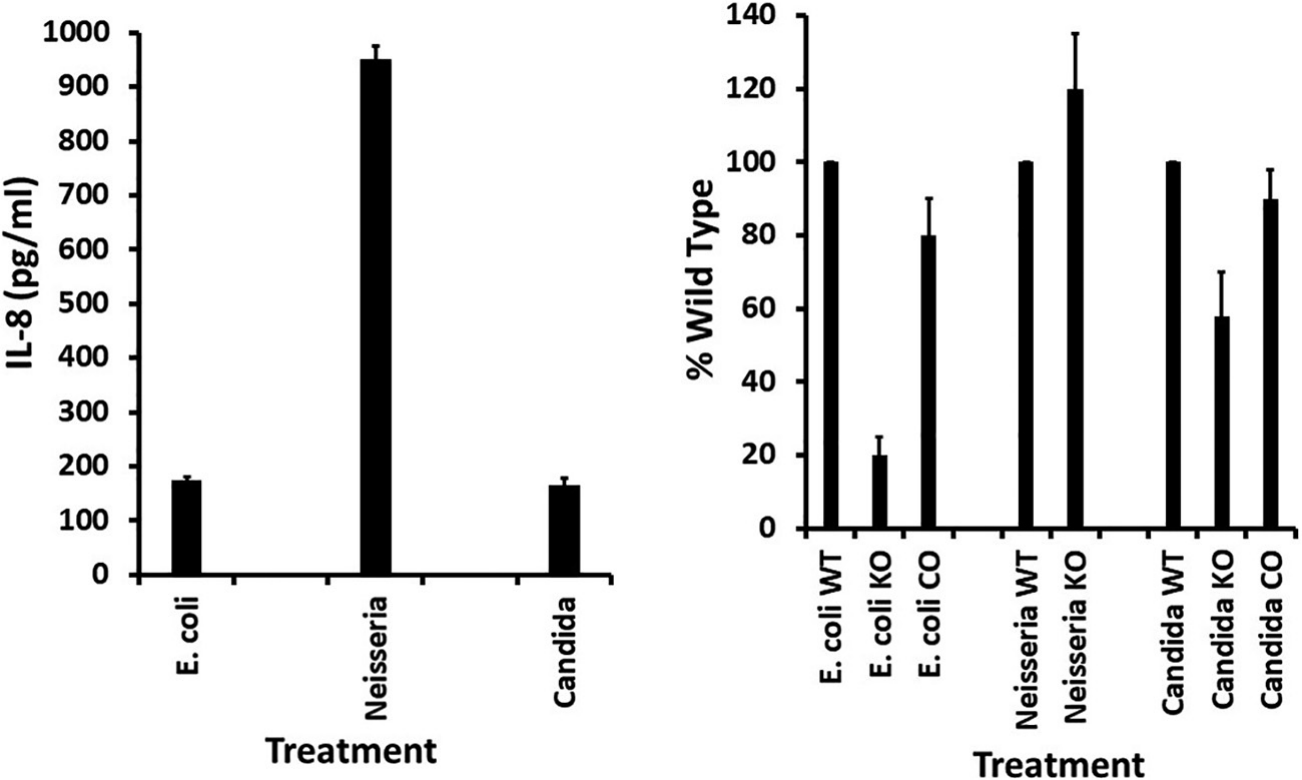
Wild-type (WT) human vaginal epithelial cells (HVECs), CD40 knockout (KO) HVECs, and CD40-complemented (CO) HVECs treated for 6 h in triplicate with various amounts of Escherichia coli, Neisseria gonorrhoeae, and Candida albicans. Left panel shows IL-8 ± standard deviations. Right panel shows percent wild type of HVEC CD40 knockout cells (KO) and CD40-complemented HVEC knockout cells (CO) treated with the optimal dose of strains that gave the peak IL-8 response by wild-type cells.

We tested two Gram-negative bacteria and Candida albicans for stimulation of IL-8 production by HVECs and the percent use of CD40 as a receptor (Fig. 4). Both Gram-negative pathogens stimulated HVECs to produce IL-8, with Neisseria gonorrhoeae (5 × 10^9^ CFU/mL) stimulating to a much greater extent (Fig. 4, left panel). Since our HVEC line lacks TLR-4 and major histocompatibility complex II molecules, the stimulation was not due to interaction with these molecules. The other known SAg receptors are T cell receptors; it is unlikely that HVECs have these receptors. Candida albicans (4 × 10^8^ CFU/mL) stimulated HVECs to produce IL-8 to the same extent as Escherichia coli (2 × 10^9^ CFU/mL) (Fig. 4, left panel). For Escherichia coli, 80% of the response depended on interaction with CD40, as the response of CD40 knockout cells was only 20% of wild-type HVECs (Fig. 4, right panel). This response was restored by complementation of the knockout HVECs with CD40.

The IL-8 response of HVECs to Neisseria gonorrhoeae did not depend on CD40, as knockout cells showed the same response as wild-type cells (Fig. 4, right panel). Thus, there was no reason to evaluate the complemented HVECs for this organism since the CD40 knockout HVECs showed similar IL-8 induction as wild-type HVECs.

The response of HVECs to Candida albicans depended 40% on the presence of CD40, as shown by the 40% reduction in IL-8 in the knockout cells (Fig. 4, right panel). The response was complemented by addition of CD40 into the knockout cells.

## DISCUSSION

Many bacterial and *Candida* pathogens cause harmful chemokine production on mucosal surfaces, such as the vaginal mucosa (20, 22, 35). We have previously referred to this as outside-in signaling to disrupt the mucosal barrier, leading to downstream infection (35–37). While in the present paper we focused on IL-8 production by HVECs, the response to pathogens more broadly affects chemokine production by these human cells, including that of IL-8, MIP-3α, and IL-33 (20, 22, 23).

S. aureus and Streptococcus pyogenes are the two common causes of TSS (38, 39). S. aureus strains, usually USA200 (CC30), which produce the SAg TSST-1 cause 100% of menstrual TSS associated with vaginal infection (38–40). Streptococcus pyogenes strains are the major cause of streptococcal TSS, most often associated with infections through breaks in the skin (7, 8). However, highly fatal cases have been associated also with vaginal infections with Streptococcus pyogenes, usually in the late second and early third trimesters (41). Approximately 85% of streptococcal TSS cases are associated with the SAg SPE A (38, 42). These pathogens also produce cytolysins which can kill HVECs when present in high concentrations, contributing to SAg transport across barriers.

Previously, we have shown that the SAgs, TSST-1 and SEB, use CD40 as their sole receptor on HVECs for production of chemokines (22). We confirmed the findings for TSST-1-induced IL-8 in this paper and also showed that SPE A stimulates HVECs to produce IL-8, dependent on CD40. In contrast, the inflammatory receptor for the TSST-1, SEB, and SPE A SAgs on keratinocytes appears to involve at least one other receptor as well as CD40, this other receptor involving the glycoprotein gp130 (23).

It has also been shown that group B streptococci, viridans streptococci, and enterococci occasionally cause TSS and severe invasive diseases (9, 10, 39, 43). SAg characterization has not been thoroughly, or at all, investigated for these strains. However, our prior studies have shown that Enterococcus faecalis strain OG1ssp(pINY1801) produces cell-associated factors that mimic the actions of SAgs (34). Additionally, we partially characterized a SAg associated with a group B streptococcal strain associated with TSS (10). It is possible that uncharacterized SAgs or SAg-like molecules produced by these strains accounted for some of the IL-8 induction in HVECs in this study.

However, it is clear that HVECs are stimulated significantly through CD40 by the various Gram-positive, Gram-negative, and *Candida* pathogens studied in this work. However, since these pathogens showed variable stimulation of IL-8 production in CD40 knockout cells, from only 20% to 100%, there are other receptors engaged. For example, the SAg TSST-1 dysregulates only approximately 600 genes in HVECs, including those for many chemokines, whereas TSS S. aureus strains dysregulate the same genes but to a total of several thousand genes (20). We know that HVECs lack TLR-4, but the cells do contain TLR-2, and thus, some stimulation by many of the pathogens we studied could be through lipoteichoic acid-peptidoglycan engagement with TLR-2. Several studies have shown direct engagement of group B streptococci with lung epithelial cells through cytolysins (14) and with innate immune cells through TLRs (15, 44). The staphylococcal cytolysin, alpha-toxin, and Streptococcus pyogenes streptolysin O are cytotoxic to HVECs at high concentrations, as we observed in these studies. However, at reduced concentrations, as would be present in dilutions of S. aureus MNPE cultures or Streptococcus pyogenes strains, these cytotoxins are likely to stimulate IL-8 production (28, 33).

We observed that two normal vaginal flora microbes, an Enterococcus faecalis strain producing reutericyclin (3) and L. crispatus, did not stimulate IL-8 production. Reutericyclin has previously been shown to be anti-inflammatory as well as broadly antimicrobial (45, 46). These data collectively show that not all bacteria on mucosal surfaces induce harmful chemokine responses. It should be noted that we have also observed that 10^8^ latex beads/mL do not stimulate chemokine production (3).

It is potentially of greatest importance that many vaginal pathogens, in part or entirely, use CD40 as the receptor on HVECs. Epithelial cells dominate the cell types present vaginally, as opposed to more-recognized types of immune cells (21). This raises the possibility that a small-molecule inhibitor of pathogen interaction with CD40 could be found. In this way, it is possible that reductions in vaginal infections and concomitant pathogenesis could be achieved. CD40 is a trimeric immune costimulatory molecule that is important systemically for immune responses (22, 47). However, there is little evidence that vaginal mucosal cell CD40 is critical for protective immune responses. Indeed, our studies suggest that interaction of pathogens with CD40 is more likely to be harmful (22). Thus, we think investigations should be initiated to identify such inhibitors that block pathogen-CD40 interactions. This of course depends on the small molecules having inhibitor activity and generally in the nanomolar range. We have already tested a monoclonal antibody that was designed to block CD40 interaction with its T lymphocyte ligand. Rather than blocking inflammation due to SAg interaction with CD40, the monoclonal antibody enhanced inflammation due to SAg, most likely due to stimulation of CD40 (22).

As a final critical point to be made, it is well recognized that TSST-1, and this TSST-1-producing S. aureus, causes menstrual TSS (40). It is also well known that group B streptococci cause neonatal sepsis and meningitis (9). However, it is not nearly as well known that approximately 2% of women have Streptococcus pyogenes vaginally. This makes these women potentially susceptible to streptococcal TSS associated with such vaginal colonization, often occurring during pregnancy or at delivery (41).

## MATERIALS AND METHODS

### Microbes.

S. aureus MN8 was obtained from the eighth menstrual TSS patient in Minnesota in 1980. The strain is a commonly used USA200 (CC30) organism (20, 22, 23, 48, 49). The organism produces TSST-1, has the enterotoxin gene cluster of SAgs (26), and has a mutation in the alpha-toxin gene, causing the strain to produce at least 50-fold less alpha-toxin (25). S. aureus MNPE was obtained from a fatal case of postinfluenza TSS as described previously (24). The organism is a USA200 (CC30 strain) that produces TSST-1, has the wild-type alpha-toxin gene (typical of skin strains), and contains the enterotoxin gene cluster of SAgs (26). A knockout strain of S. aureus MN8 in the TSST-1 gene was prepared as described previously (26). This strain has growth characteristics typical of S. aureus MN8, except the strain does not produce TSST-1. S. epidermidis was a skin isolate from a healthy individual (50). All strains of low passage number are maintained at −80°C in the Schlievert laboratory.

Group A streptococcal strain T25_3_ was provided originally by Dennis Watson, University of Minnesota (now deceased), as a lyophilized stock culture. The Schlievert laboratory cured the strain of an endogenous bacteriophage, giving rise to strain T25_3_(cured) (30, 31). The bacteriophage T12, which carries the gene for streptococcal pyrogenic exotoxin A (SPE A), was inserted into strain T25_3_(cured) to give rise to T25_3_(cured)T12 (30, 31). Strain 594 (SPE A positive) was obtained from Nauciel et al. (29). All strains are maintained as low-passage-number −80°C stocks in the Schlievert laboratory.

Group B streptococcal strain MNSI was from an infant with neonatal sepsis and meningitis (9). Two Enterococcus faecalis strains were used in this study. One was a vaginal isolate from a woman with a pure culture of this organism. The organism produces the broadly antimicrobial and anti-inflammatory molecule reutericyclin (3). The second isolate was submitted to the Schlievert laboratory as a strain from a patient with nonmenstrual TSS.

Escherichia coli (Watson) was originally a clinical isolate from a urinary tract infection, as generously provided by Dennis Watson, University of Minnesota (now deceased) (51). Neisseria gonorrhoeae was a clinical isolate kindly provided by Michael Apicella, University of Iowa (now retired). Candida albicans was a generous gift from Daniel Diekema, University of Iowa. All strains are maintained as low-passage-number cultures at −80°C.

### Superantigens.

TSST-1 and SPE A, used as positive controls for interaction with CD40, were prepared from clones in S. aureus strain RN4220, a strain which does not appear to secrete SAgs (22, 52–54). In the 1980s, the United States Recombinant DNA Advisory Committee (RAC) gave approval for the Schlievert laboratory to clone SPE A in S. aureus RN4220 based on the relatedness of SPE A to both SEs B and C. The RAC agreed with Schlievert that SPE A and SEs B and C shared a common ancestor in S. aureus, with the SPE A gene most likely transferred to S. pyogenes by bacteriophages.

### Microbial culture.

All S. aureus strains, Escherichia coli, and Candida albicans were cultured to stationary phase in Todd-Hewitt broth (THB; Difco, Detroit, MI) at 37°C with 200-rpm shaking. Streptococci and enterococci were cultured to stationary phase in THB at 37°C in the presence of 5% CO_2_ as stationary cultures. The stationary-phase organisms were used directly in experimentation. For those microbes that produced cytotoxins, dilutions of the stationary-phase cultures were made in THB. Neisseria gonorrhoeae was cultured on chocolate agar plates for 24 h at 37°C in the presence of 5% CO_2_. The organisms were gently scraped off the plates and used directly for experimentation.

### Human vaginal epithelial cells (HVECs).

The wild-type, CD40 knockout, and CD40-complemented knockout cells have been described previously (22). The cells were cultured at 37°C in the presence of 5% CO_2_ in keratinocyte serum-free medium until nearly confluent. The cells were then split and cultured until confluent in 96-well microtiter plates.

### Experimentation.

All experiments used triplicate replicates, and experiments were independently repeated at least one additional time. Microbes were cultured as given above. The HVECs were cultured in 180 μL to confluence, and medium was changed in 5% CO_2_ at 37°C. Then, 20 μL of each bacterial culture was added to each well, and wells were incubated for 6 h. Subsequently, the samples were frozen at −20°C for 24 h to release any intracellular chemokine. IL-8 was measured with use of Quantikine kits as purchased from R&D Systems, Minneapolis, MN.

### Statistics.

Means ± standard deviations were determined. For data presented in Fig. 1 and 2, statistical significance of means of IL-8 between S. aureus MN8 wild type versus MN8Δ*tst* and S. pyogenes T25_3_(cured)T12 versus T25_3_(cured), the analyses were performed on a fee-for-service basis by the Director of the Biostatistics Core in the University of Iowa, Carver College of Medicine, Clinical and Translational Science Award Program, Patrick Ten Eyck. The analyses included the following. We utilized the generalized linear modeling (GLM) framework to test for between-group differences in the outcome measure at different dilutions of microbes. Main effect predictors included group, dilution, and the interaction effect (group × dilution). To account for the right-skewed distribution in the outcome measure, we specified a log-link function and assessed the between-group mean ratio. Tests with *P* values of <0.05 were considered statistically significant.
